# Photosynthetic symbiotic therapy

**DOI:** 10.18632/aging.101796

**Published:** 2019-01-25

**Authors:** Hanjay Wang, Matthew A. Wu, Y. Joseph Woo

**Affiliations:** 1 Department of Cardiothoracic Surgery, Stanford University School of Medicine, Stanford, CA 94305, USA

**Keywords:** oxygen-producing biomaterials, photosynthesis, ischemia, hypoxia, symbiosis

**Figure 1:**
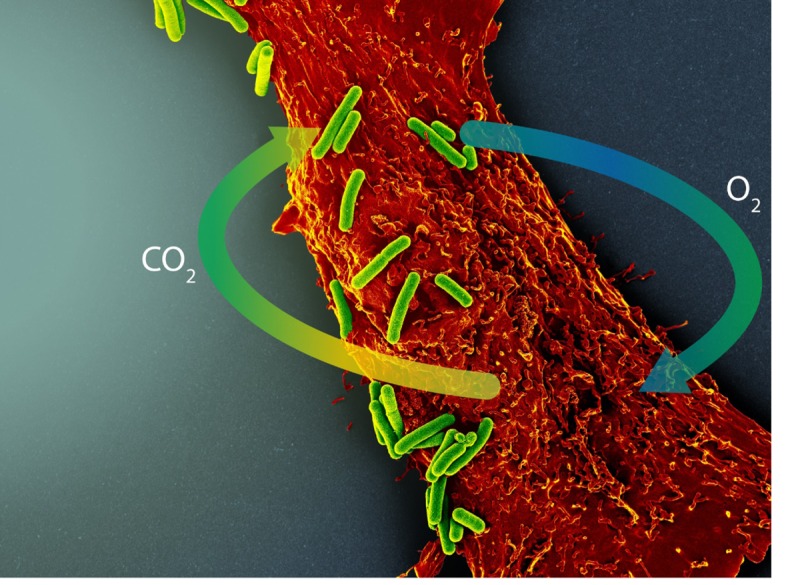
Schematic example of photosynthetic symbiotic therapy, derived from a false-color scanning electron micrograph showing multiple cyanobacteria (*S. elongatus*, green) in co-culture with a single rat cardiomyocyte (red). In the presence of light (image left), the photosynthetic cyanobacteria recycle CO_2_ produced by cardiomyocytes, generating O_2_ for continued aerobic respiration.

Engineered O_2_-producing biomaterials represent an emerging field with enormous potential to address tissue ischemia and hypoxia without revascularization. The clinical applications span nearly the entire domain of medicine and include the areas of tissue engineering and regeneration, organ preservation, wound healing, diabetic microvascular disease, and cardiovascular, cerebrovascular, and peripheral vascular disease. Nature, however, evolved the most elegant O_2_-producing biomaterial 3.5 billion years ago in the form of photosynthetic cyanobacteria, which are responsible for the relative abundance of O_2_ in Earth’s atmosphere today. These ancestors of the chloroplast convert CO_2_ and water into O_2_ and glucose using light as an energy source. Recently, teams have begun to engineer symbioses between cyanobacteria or other photoautotrophic algae and heterotrophic cells such as those of mammals. In this relationship, the photosynthetic microorganism recycles CO_2_ produced by heterotrophic cellular respiration and generates O_2_ that helps sustain the heterotrophic partner (Figure 1). Here, we summarize the current state of photosynthetic symbiotic therapy and the steps taken toward clinical translation.

The first use of a photosynthetic microorganism to remedy tissue hypoxia *in vivo* was reported by Yamaoka and colleagues in 2012 [1]. By placing a gas-permeable pouch containing a light-emitting diode and the photosynthetic microalga *Chlorella vulgaris* in the perfluorocarbon-filled peritoneal cavity of hypoventilated rats, the team demonstrated that *Chlorella* could supplement gas exchange in rats with respiratory insufficiency. Yamaoka et al. also explored the use of photosynthetic symbiosis to enhance the viability of heterotopically-transplanted rat pancreases harvested 3 hours after cardiac death. The team demonstrated that a majority of diabetic recipient rats receiving pancreases stored in traditional cold preservation solution for 30 minutes exhibited severe glucose dysregulation and died within 5 hours after surgery. All rats receiving pancreases stored similarly but with *Chlorella* in gas-permeable bags at mild hypothermia (22°C), however, had normal blood glucose levels and survived beyond 1 week after surgery.

More recently, Cohen et al. described the first use of photosynthetic symbiosis to treat cardiac ischemia [2]. Following ligation of the left anterior descending coronary artery in rats, the cyanobacterium *Synechococcus elongatus* was injected into the ischemic territory. Under surgical lighting, rats receiving cyanobacteria exhibited markedly improved myocardial O_2_ levels within 10 minutes, in association with recovery of cellular metabolic activity and enhanced cardiac function compared to saline or dark controls. Using a rat ischemia-reperfusion model, Cohen and colleagues also demonstrated that photosynthetic therapy limited only to the duration of open chest surgery yields durable long-term benefits in cardiac function at 4 weeks after ischemic injury.

Other groups have also investigated symbiotic relationships between photosynthetic microorganisms and mammalian cells using hypoxic, *in vitro* co-culture environments. These include studies showing that photosynthesis by *Chlamydomonas reinhardtii* can attenuate the molecular response of mouse fibroblasts to hypoxia [3], and that photosynthesis by *Chlorococcum littorale* can switch rat cardiomyocyte metabolism from anaerobic to aerobic pathways [4].

Direct inoculation of host tissues with microorganisms in solution risks rapid loss of the symbiotic microbes. Although no *in vivo* study has yet to demonstrate a significant immune response against a photosynthetic symbiont (i.e. against *S. elongatus* or *C. reinhardtii* in zebrafish, mouse, or rat models) [2,5–8], delivery via a bioengineered construct nevertheless reduces the rate of cell dispersal. To this end, Egaña and colleagues have conducted impressive pioneering work on the development of photosynthetic algae-seeded scaffolds. Using an FDA-approved collagen-based scaffold, Egaña’s team demonstrated that *C. reinhardtii* seeded within the scaffold were able to photosynthesize effectively and even proliferate [3]. Moreover, *C. reinhardtii* co-cultured with murine fibroblasts within the scaffold were able to supply the fibroblasts with O_2_ in hypoxic conditions. In a subsequent study, Egaña’s team improved the translatability of their delivery system by encapsulating *C. reinhardtii* in a biocompatible fibrin-based hydrogel before scaffold seeding, thus minimizing cell scattering [5]. These hydrogel scaffolds were safely applied as skin grafts to treat full-skin defects in mice [5,6].

In contrast, Haraguchi et al. embedded *C. littorale* into multilayer cell sheets composed of rat cardiomyocytes [4]. As a result, engineered cardiac tissues could be made 4-fold thicker (160 μm) and still remain viable without any associated vascularization when maintained in photosynthetic symbiotic co-culture. Finally, it is also possible to generate viable endosymbiotic chimeric tissues, as shown by two separate teams that injected photosynthetic microorganisms into developing zebrafish embryos [7,8]. Indeed, Agapakis et al. even demonstrated that *S. elongatus* could be engineered to express the protein machinery needed to directly invade and grow within cultured mammalian cells including macrophages [7]. Altogether, these studies illustrate the potential of cyanobacteria and microalgae to contribute to the development of photosynthetic engineered tissues.

Thus far, photosynthetic symbiotic therapies have not taken full advantage of the immense genetic adaptability of cyanobacteria and microalgae. While Chavez et al. engineered *C. reinhardtii* to express and secrete vascular endothelial growth factor (VEGF), resulting in O_2_ delivery as well as neoangiogenesis when delivered into zebrafish and rat tissues *in vivo* [6], nearly all attempts to treat tissue ischemia or hypoxia using photosynthetic symbiosis have focused solely on gas exchange alone. Future studies should aim to expand the arsenal of clinically useful compounds produced by the photosynthetic symbiont and thereby augment its therapeutic potential.

Overall, photosynthetic symbiosis represents a valuable untapped strategy for the development of novel engineered O_2_-generating biomaterials. Indeed, a solution for many of humanity’s modern medical challenges may ultimately evolve from reengineering Nature’s oldest oxygenic microorganisms.
